# Perioperative multidisciplinary implementation enhancing recovery after hip arthroplasty in geriatrics with preoperative chronic hypoxaemia

**DOI:** 10.1038/s41598-019-55607-8

**Published:** 2019-12-16

**Authors:** Liang He, Ruqiang Zhang, Jianwei Yin, Han Zhang, Wuming Bu, Fang Wang, Furong Zhang

**Affiliations:** Department of Anesthesiology, Yan’an Hospital of Kunming City, Kunming Medical University, Kunming, 650051 P.R. of China

**Keywords:** Outcomes research, Risk factors

## Abstract

We investigated risk factors for postoperative serious adverse events (SAEs) in elderly patients with preoperative chronic hypoxaemia undergone total hip arthroplasty (THA) or hemiarthroplasty and performed an implementation to modify and improve clinical outcome. A retrospective medical record review was performed to identify geriatric patients who receiving THA or hemiarthroplasty at a single university teaching hospital, Kunming, Yunnan, China between January 2009 and August 2017. Total of 450 elderly patients were included in the study. Data were collected on baseline characteristics, detailed treatments, and adverse events. Univariate and multivariate logistic regression analysis were used to identify risk factors for SAEs. In multivariate regression analysis, a higher occurrence of general anaesthesia and multiple episodes of hypotension were associated with higher risk of SAEs (general anesthesia: odds ratio [OR] 5.09, 95% confidence interval [CI] 1.96–13.24, P = 0.001; hypotension time: OR 4.29, 95% CI 1.66–11.10, P = 0.003). After the multidisciplinary implementation, the postoperative length of stay was decreased from 15 days to 10 days (P < 0.0001); incidence of SAEs was decreased from 21.1% to 7.0% (P = 0.002), and the all-cause mortality rate within 30 days decreased from 4.6% to 1.0% (P = 0.040). Our observational study demonstrated that an increasing application of general anaesthesia and longer time of hypotension were associated with an increased risk of postoperative SAEs in patients after THA or hemiarthroplasty. Additionally, optimizing stable haemodynamics under higher application of combined-spinal epidural anaesthesia was associated with improved outcome up to 30 days after THA or hemiarthroplasty.

## Introduction

Total hip arthroplasty (THA) or hemiarthroplasty is performed in a wide variety of patients, ranging from those requesting surgery to facilitate their highly active lifestyle to those who require surgery in order to perform routine activities of daily living^1^. Attenuation of pain and improvement in quality of life for patients suffering severe hip osteoarthritis or fracture are achieved^1,2^. However, decreasing quality of life is associated with patient- and surgery-related factors^3,4^. Aging, anaesthesia, perioperative hypoxaemia, hypotension and anaemia appear to be associated with the development of poor outcomes including delirium in geriatric patients after orthopaedic surgery^5,6^. Surprisingly, except for our own limited conclusion^7^, few data have tested the associations between anaesthesia, preoperative chronic hypoxaemia, high altitude, age, perioperative management, and postoperative serious adverse events (SAEs) including pulmonary embolism, malignant arrhythmia, intensive care unit admission) after THA or hemiarthroplasty. In view of the above mentions and increasing international recognition that perioperative management does affect outcomes^3,8^, we designed a multidisciplinary perioperative management implementation protocol for THA or hemiarthroplasty.

## Methods

The local Institutional Review Board for Clinical Investigations of Yan’an Hospital of Kunming City approved the study and put forward the protocol presented as later in details. The authors confirm that all methods were performed in accordance with the relevant guidelines and regulations by including a statement in the methods section to this effect. For the retrospective review of electronic medical record, the written informed consent was not needed. In view of unavoidable environmental conditions including high altitude (above 1850 m), complicated comorbidities including moderate hypoxaemia (defined as arterial partial pressure of oxygen, PaO_2_: mild, 55–70 mmHg; moderate, 40–55 mmHg, severe, less than 40 mmHg, with air inhalation), most elderly patients have to be transferred from other hospital(s) into this one. Therefore, when it comes to preoperative evaluation for patients demand of THA or hemiarthroplasty, most of them in this hospital were with American Society of Anesthesiologist (ASA) physical status of III or IV, and some of them with language and communication barriers.

At baseline, there was no written guideline for the acceptable level of preoperative chronic hypoxaemia (CH) should be managed before operation. Some domestic clinical experts recommend that patients with PaO_2_ less than 60 mmHg should be taken into seriously, particularly to those with PaO_2_ less than 50 mmHg, but it is lack of necessary documented files and references in THA or hemiarthroplasty. Although there was an agreement that patients may be referred to the respiratory consultation, which was not used for every patient, because of the maximum tolerance of preoperative CH without clear identification.

**Figure 1:**
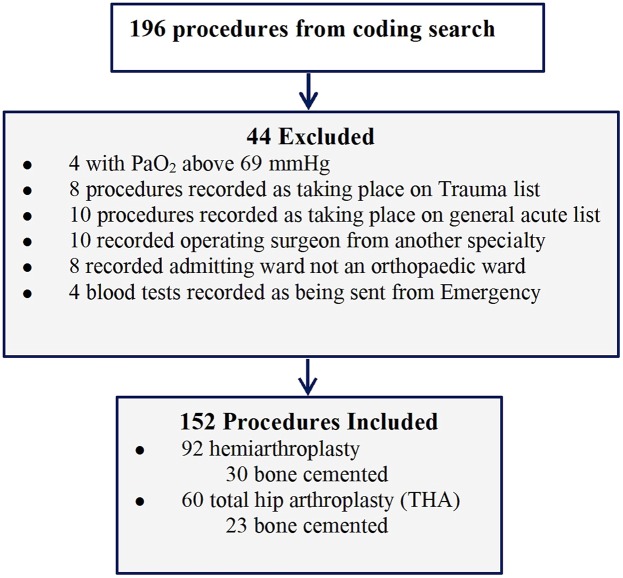
Search and excluded records.

There was also no specific guideline on intraoperative management measures. So, study was performed as following in details. Firstly, we retrospectively researched this hospital paper documents for all THA or hemiarthroplasty coded between January 2009 and June 2012 (details in Fig. 1). These databases are subject to serve as a control.

Patients who had more than lateral THA or hemiarthroplasty in the period concerned were considered once, that is, the unit analyzed was the patient with a unique in-hospitalization number. We collected gender, age, preoperative hemoglobin (Hb), ASA status, PaO_2_, SpO_2_, and comorbidities. The postoperative data were Hb, serious adverse events (SAEs, including pulmonary embolism, malignant arrhythmia needing cardiologist consultation, and delirium), length of stay (LOS), incidence of ICU admission, and mortality before discharge.

**Figure 2:**
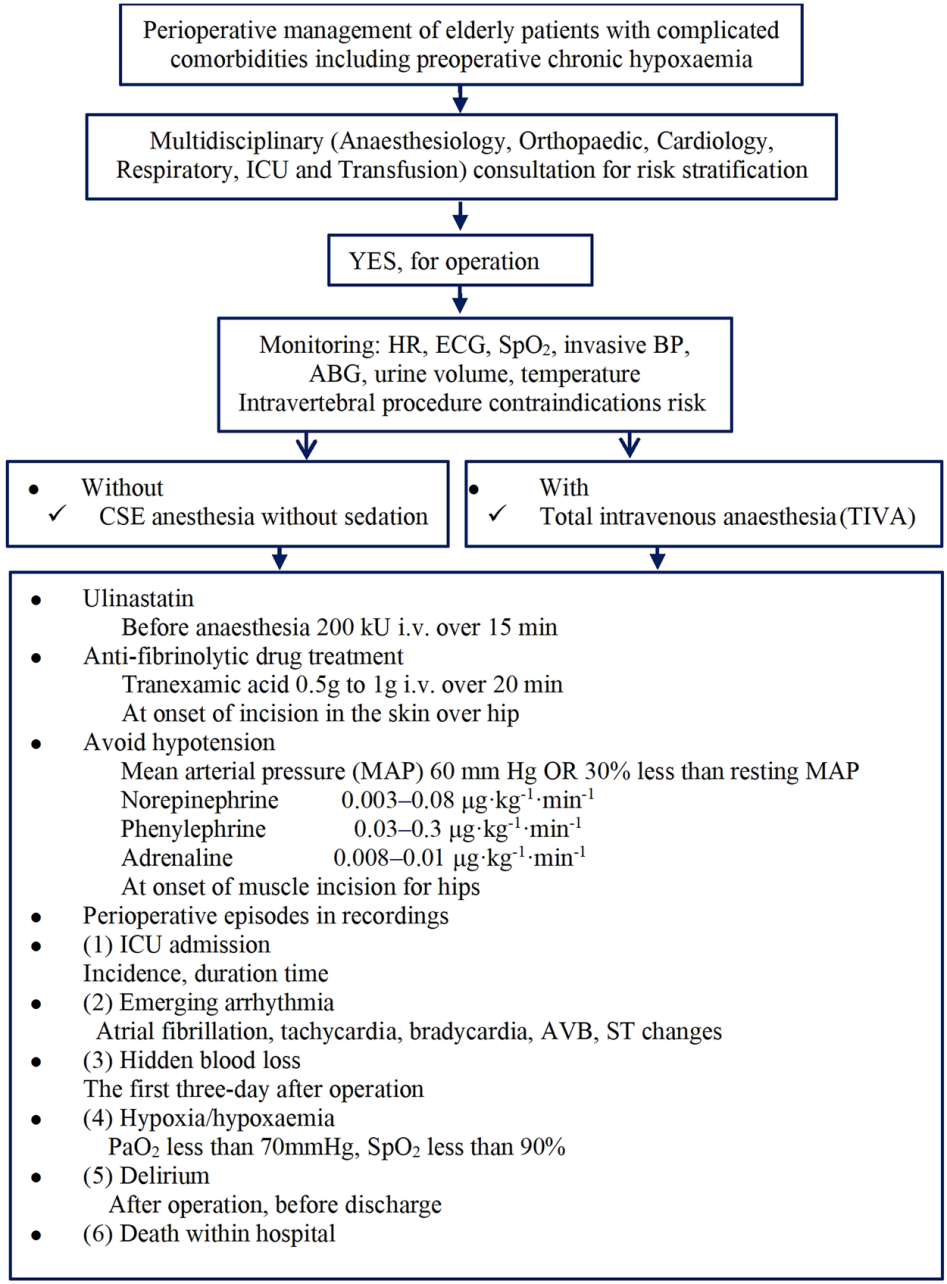
Perioperative multidisciplinary management implantation.

A multidisciplinary perioperative implementation protocol used for THA or hemiarthroplasty is explained as followings: (1) For plausible procedure, demographic characteristics of geriatric patients with complicated comorbidities including preoperative chronic hypoxaemia are in consultation with departments of Anaesthesiology, Orthopaedic surgery, Cardiology, Respiratory, ICU and Transfusion to classify risk stratification. (2) If “YES for operation”, standard monitoring of HR, ECG, SpO_2_, invasive BP, ABG, urine volume, temperature is in performance. Elderly patients in absence of intravertebral procedure contraindications are received procedure under combined-spinal epidural (CSE) anesthesia without any other anesthetics for sedation, if not, total intravenous anaesthesia (TIVA) was performed. Thromboprophylaxis and discharge criteria are based on local protocols applicable to all inpatients. Wound drain is universal through a negative pressure drainage ball. (3) In addition to treating hypoxaemia, hypotension in geriatric patients, administration of ulinastatin at dosage of 200–300 kU intravenously over 15 min before CSE or general anaesthesia. Tranexamic acid (TXA), an anti-fibrinolytic drug, 0.5–1 g i.v. over 20 min is administrated at onset of incision of the skin over the hip. To avoid a bolus of any vasopressor agents on fluctuation of blood pressure, combination of continuous intravenous infusion with norepinephrine at 0.003–0.08 μg·kg^−1^·min^−1^, phenylephrine 0.03–0.3 μg·kg^−1^·min^−1^, adrenaline 0.008–0.01 μg·kg^−1^·min^−1^ (particular to bone cemented demands) was at the time of muscle isolation from hip. (4) Perioperative episodes are in recordings: ICU admission (incidence, duration time); emerging arrhythmia (atrial fibrillation, tachycardia, bradycardia, AVB, ST changes); hidden blood loss; hypoxia/hypoxaemia (PaO_2_ less than 70 mmHg, SpO_2_ less than 90%); delirium (after operation, before discharge); and death within hospital, details in Fig. 2.

Logistic regression analyses for baseline data in control was conducted. The effect of age, sex, preoperative Hb, and intraoperative hypotension on outcomes of SAEs, LOS and all-caused death within 30 d after procedures were explored to identify independent risk factors in the models. Next, procedure data of primary THA or hemiarthroplasty from January 2013 (multidisciplinary management onset of October 2012) to August 2017 were collected. And then, we compared these against the retrospective controls on an intention-to-treat basis, using SPSS for Windows (Version 16.0, SPSS, Inc., Chicago, IL). In addition, binary outcomes were analyzed by Chi-square or Fisher’s exact test. According to the Kolmogorov–Smirnov test, a Gaussian distribution of continuous data was tested to be conducted by t-tests, or the Mann–Whitney tests.

## Results

### Incidence of preoperative chronic hypoxaemia

From January 2009 to August 2012, we reviewed and identified 196 patients. Forty-four were excluded (factors in Fig. 1), resulting in analysis of 152 patients for primary THA or hemiarthroplasty at this hospital, a teaching hospital of Kunming Medical University. A total of 152 patients were performed as 52 bone cemented joint and 100 without. Transfusion, SAEs, admission, and discharge were available for all patients in this study. The proportion of mild, moderate and severe hypoxaemia were 22.3% (34/152), 70.5% (107/152), and 7.2% (11/152) respectively.

### Identification of risk factors

For the candidate risk factors, performed by logistic regression with one step method, data were presented by odds ratio (OR) with 95% confidence interval (CI) as following: intraoperative hypotension (mean arterial pressure, mean arterial pressure (MAP), decreased by fraction 30% from baseline, lasting over 3 min) (OR 4.29; 95% CI 1.66 to 11.10; *P* = 0.003), gender (OR 1.38; 95% CI 0.56 to 3.41; *P* = 0.488), age (OR 1.02, 95% CI: 0.35 to 3.00, *P* = 0.974), General anaesthesia (OR 5.09; 95% CI 1.96 to 13.24; *P = *0.001), preoperative hypoxaemia (OR 0.90; 95% CI 0.34 to 2.43; *P* = 0.841), anaemia (OR 2.28; 95% CI 0.86 to 6.05; *P* = 0.099), blood transfusion (OR 1.40, 95% CI 0.44 to 4.44; *P* = 0.573), history of cardiovascular diseases (OR 0.94; 95% CI 0.40–2.19; *P* = 0.881), history of cerebral diseases (OR 2.95; 95% CI 0.99–8.81; *P* = 0.052), history of diabetes (OR 2.81; 95% CI 0.84 to 9.38; *P* = 0.093), history of pulmonary diseases (OR 1.74; 95% CI 0.36 to 8.36; *P* = 0.49), and surgery site (total/hemi-) (OR 0.59; 95% CI 0.24 to 1.47; *P* = 0.256) for SAEs, in Table 1.

**Table 1:** Variables in the Equation by logistic regression.

Possible risk factors	B	S.E.	Wald	Df	Sig.	Exp(B)	95.0% C.I.for EXP(B)	
							Lower	Upper
Gender	0.321	0.462	0.482	1	0.488	1.378	0.557	3.412
Age	0.018	0.551	0.001	1	0.974	1.018	0.346	2.999
Hypotension	1.456	0.485	9.012	1	0.003*	4.289	1.658	11.097
General anaesthesia	1.628	0.487	11.165	1	0.001*	5.094	1.96	13.238
Hypoxaemia	−0.101	0.504	0.04	1	0.841	0.904	0.337	2.427
Anaemia	0.823	0.499	2.722	1	0.099	2.277	0.857	6.054
Transfusion	0.333	0.591	0.318	1	0.573	1.395	0.438	4.442
Cardiovascular diseases	−0.065	0.433	0.022	1	0.881	0.937	0.401	2.191
Cerebral diseases	1.083	0.558	3.774	1	0.052	2.954	0.99	8.812
Diabetes	1.034	0.615	2.826	1	0.093	2.812	0.842	9.385
Pulmonary diseases	0.553	0.801	0.477	1	0.49	1.739	0.362	8.361
Surgery	−0.532	0.468	1.292	1	0.256	0.588	0.235	1.47
Constant	−2.277	1.108	4.226	1	0.04	0.103		

One step to logistic regression was used in this analysis as details: Age, 1 = no more than 70 y, 2 = 71–90 y, 3 = above 91; Hypoxemia, 1 = mild, 2 = moderate, 3 = severe; Cardiovascular diseases, Cerebral diseases, Diabetes, General anesthesia, Hypotension, Pulmonary diseases, Transfusion, 1 = Yes, 0 = No; Gender, female = 1, male = 0; Surgery, 1 = total hip arthroplasty, 0 = hemi-. *P < 0.05.

### Improvement after multidisciplinary implementation

Patient multidisciplinary management implementation was associated with shorter postoperative LOS for THA (or hemiarthroplasty) decreased from 15 (10–22) days to 10 (7–13) days, after implementation (*P* < 0.001). The SAEs ratios decreased from 21.1% (32/152) before to 7.0% (21/298) after modification (*P* = 0.002). The all-cause mortality rate within 30 days after procedure was decreased from 4.6% (7/152) before to 1.0% (3/298) after modification (*P* = 0.040). Percentages of procedure under CSE anaesthesia were increased from 32.9% (50/152) before up to 92.6% after implementation (276/298) (*P* < 0.001). Patients, after implementation, were with higher PaO_2_ (*P* < 0.001) and ratios of PaO_2_/FiO_2_ (*P* < 0.001), but lower incidence of hypoxia (*P* = 0.041), lower occurrence of ICU admission (*P *= 0.009), lower incidence of malignant arrhythmia (*P* = 0.013), lower appearance of delirium (*P* = 0.002),  and less red blood cell loss (*P* = 0.007), details in Table 2.

**Table 2:** Parameters changed after implantation programmed management.

	Before	After implantation	*P*
*N*	152	298	
Age (y), Mean ± SD	78 ± 7	79 ± 8	0.224
Female, n(%)	97(63.8)	186(62.5)	0.802
**Baseline, before anesthesia**			
PaO_2_ (mm Hg)	52 ± 7	51 ± 6	0.155
SpO_2_ (%)	87 ± 4	86 ± 6	0.078
Ratios of PaO_2_/FiO_2_ (mm Hg)	254 ± 48	259 ± 53	0.366
**Within 30 min prior to suture**			
PaO_2_ (mm Hg)	112 ± 17	141 ± 15	<0.001*
SpO_2_ < 97%, n(%)	17(11.2)	17(5.8)	0.041*
Ratios of PaO_2_/FiO_2_ (mm Hg)	274 ± 54	324 ± 60	<0.001*
**Intraoperative MAP decreased**			
by fraction <20%, n(%)	81(53.2)	244(81.8)	<0.001*
by fraction ≥30%, n(%)	51(33.6)	17(5.7)	<0.001*
**Intraoperative hypotension (min)**			
Lasting time (<3 min), n(%)	14(9.2)	15(5.2)	0.148
No less than 3 min, n(%)	37(24.3)	3(1%)	<0.001*
Red blood cell loss (ml)	157 ± 71	137 ± 65	0.007*
Anaemia, n(%)	38(25.0)	79(26.5)	0.742
Transfusion, n(%)	21(13.8)	39(13.0)	0.830
General/Epidural/CSE anaesthesia (n)	31/71/50	6/12/280	<0.001*
Cardiovascular diseases, n(%)	67(44.1)	135(45.3)	0.818
Cerebral diseases, n(%)	23(15.1)	60(20.3)	0.214
Diabetes, n(%)	18(11.8)	45(15.1)	0.382
Pulmonary diseases, n(%)	24(15.8)	48(16.1)	0.929
postoperative SAEs, n(%)	32(21.1)	28(9.4)	0.002*
ICU admission, n(%)	27(17.8)	21(7.0)	0.009*
Malignant arrhythmia, n(%)	7(4.6)	2(0.7)	0.013*
Delirium, n(%)	10(6.5)	3(1.0)	0.002*
postoperative LOS (d), Median (IQR)	15(10–22)	10(7–13)	<0.001*
All-caused death within 30d (n)	7	3	0.040*

FiO_2_, fraction of inspired oxygen; ICU, intensive care unit; IQR, interquartile; N, number; PaO_2_, arterial partial pressure of oxygen; LOS, length of stay; SAEs, serious adverse events; SpO_2_, oxygen saturation (pulse oximetry); ^a^Value of Mann-Whitney U; *P < 0.05. Continuous data were presented by Mean with standard deviation (SD) (Mean ± SD), non-continuous data were presented by Median with interquartile (Median with IQR).

## Discussion

Our primary process measure was the proportion of elderly patients presenting for elective THA (or hemiarthroplasty) with preoperative chronic hypoxaemia. The proportion of patients who experienced serious adverse events (SAEs) was our primary outcome. Secondary outcomes were postoperative length of stay (LOS) and death. Obviously, a successful and effective multidisciplinary implantation program consists of multiple disciplinary team (including departments of Anaesthesiology, Orthopaedic surgery, Cardiology, Respiratory, ICU and Transfusion), optimizing stable haemodynamics under anaesthesia, pretreatment with tranexamic acid (TXA) 0.5 to 1 g i.v. over 20 min, and combination of continuous intravenous infusion of vasopressor agents of norepinephrine, phenylephrine, and adrenaline in demands. This quality improvement report, for the first time, to our knowledge, describes the design of a patient multidisciplinary management programmed following a combination of local experienced data, national guidelines, and international opinion for patients with preoperative chronic hypoxemia in high altitude areas. Systematic approaches to improving patients’ oxygenation index (ratios of PaO_2_/FiO_2_) and optimizing stable haemodynamic (lower fraction of MAP decreased by baseline) was associated with lower incidence of ICU admission, intraoperative hypotension, and SAEs, shorter LOS, less red blood cell loss and a reduction within 30-day all-caused death after primary elective THA or hemiarthroplasty. For 30-day mortality after procedure, 3 of 7 in control group, and one of two in implementation died of pulmonary embolism secondary to bone cement response syndrome within 2 days after procedure; the rest were died after discharge, but for lack of available data, the reason and time of death were unclear (details were not reported).

Compared to those of previous strategies, the use of multidisciplinary implantation program is associated with reduced mortality and incidence of SAEs in geriatric patients, with preoperative CH, undergoing THA or hemiarthroplasty. Intraoperative hypotension increased the incidence of SAEs over 4-fold in this observational study, but not in all-caused short-term mortality. There is controversy in intraoperative hypotension and adverse outcome after noncardiac surgery^9,10^. That MAP values decreasing more than 30% from baseline was associated with higher risk of postoperative ischemic stroke^11^. And when the MAP was less than 60 mmHg, patients may suffer from acute kidney injury after noncardiac surgery^12^. It suggested that reduction in SBP more than 50% from baseline lasting more than 5 min increased incidence of myocardial damage^13^. Therefore, an intraoperative blood pressure stability without fluctuations suggest improved outcomes in the present cohort study^14^.

Ulinastatin, a human urinary trypsin inhibitor, acting as immunomodulation, is widely used in sepsis, acute pancreatic^15^, and multiple organ dysfunction syndromes^16^. Several studies have reported that ulinastatin administration does inhibit pulmonary ischemia reperfusion injury, oxidant-induced endothelial hyperpermeability and apoptosis^17,18^. Although ulinastatin administration, enhanced ratio of PaO_2_/FiO_2_ in this retrospective cohort study, we did not have direct evidence to verify pulmonary hypoxaemia injury and lung capillary hyperpermeability and apoptosis in this study, so we cannot determine whether ulinastatin administration improved oxygenation by inhibiting inflammation reaction or immunomodulatory strategy. Further study regarding the mechanism of ulinastatin administration on the pulmonary capillary system may be needed.

While TXA, one of the most commonly used antifibrinolytic drugs after the restriction of aprotinin use in 2008, inhibits the conversion of plasminogen to plasmin and decrease the degree of fibrinolysis^19^, resulting less blood loss and lower incidence of transfusion in hip and knee arthroplasty^20^. To avoid extensive blood loss, given aging, hypoxaemia, high grade with ASA class III or IV, comorbidity of cardiocerebrovascular diseases, minor dosage of TXA were used in this implantation program management. Then, the amount of blood loss not incidence of perioperative transfusion was reduced to some extent, and there were not associated complications. Meanwhile, Whiting and colleagues from Mayo Clinic found TXA seemed safe and effective in about 1000 patients with severe medical comorbidities following data of a retrospective review^21^. However, a larger prospective trial is warranted to confirm results presented.

When it comes to anaesthesia techniques, in our previous practice, we found CSE anaesthesia supervisor to general anaesthesia in the geriatric patients with preoperative chronic hypoxaemia undergoing THA or hemiarthroplasty^22^. It was similar to those in retrospective analysis with large sample size^23,24^. Obviously, application of CSE anesthesia is popular in our hospital^7^. However, if ultrasound equipment is in hand and ultrasound-guided nerve block is skilled, there should be no reason for peripheral nerve block to conduct anaesthesia for geriatric patients with complicated comorbidities in high risk stratification^25^ for CSE or general anaesthesia.

## Limitations

This observational cohort study has some weaknesses. Firstly, the data collected are observational. The observed improvements may be altered by the study itself, rather than the effects of multidisciplinary management (Hawthorne effect, a research project from 1927 to 1932 of the Hawthorne plant of the Western Electric Company in Cicero, Illinois.). Secondly, given a comprehensive management and relatively smaller sample size, we also cannot comment on what the relative contributions of ulinastatin, TXA, vasoconstrictor, procedure with modification and the intraoperative measures were. Thirdly, ultrasound-guided nerve block technique is available and effective^25,26^, but not universally applicated in these patients with high-risk. At last, we collected no data on surgeon and anesthesiologist, surgical approaches with modification, or the cost-effective resource use during operation, since these are not available into this hospital databases. Also, compared with randomized controlled clinical trial, the confounding and bias is possible.

## Conclusions

We conclude that general anaesthesia and intraoperative hypotension are independent predictors of poor outcomes after primary THA or hemiarthroplasty in routine practice in China and that CSE anaesthesia and stable haemodynamics is feasible and necessary. The introduction of a patient multidisciplinary management programmed was associated with a reduction in ICU admission, blood loss, delirium, malignant arrhythmia and improvements in patient outcome, postoperative LOS and all-caused death within 30d were both decreased. While, randomized trials are necessary to determine whether multidisciplinary management programmed improves patient outcomes other than hypotension or anesthesia methods. However, high-quality prospective studies are warranted to confirm these findings and to establish evidence-based clinical guidelines.

## Data Availability

The datasets generated during and/or analyzed in this study are available from the corresponding author upon reasonable requests.
